# Parabens Increase Sulfamethoxazole-, Tetracycline- and Paraben-Resistant Bacteria and Reshape the Nitrogen/Sulfur Cycle-Associated Microbial Communities in Freshwater River Sediments

**DOI:** 10.3390/toxics11040387

**Published:** 2023-04-18

**Authors:** Chu-Wen Yang, Wei-Chen Lee

**Affiliations:** Department of Microbiology, Soochow University, Taipei 111002, Taiwan

**Keywords:** microbial communities, paraben-resistant bacteria, antibiotic-resistant bacteria, freshwater river sediments

## Abstract

**Backgrounds** Parabens are pollutants of emerging concern in aquatic environments. Extensive studies regarding the occurrences, fates and behavior of parabens in aquatic environments have been reported. However, little is known about the effects of parabens on microbial communities in freshwater river sediments. This study reveals the effects of methylparaben (MP), ethylparaben (EP), propylparaben (PP) and butylparaben (BP) on antimicrobial-resistant microbiomes, nitrogen/sulfur cycle-associated microbial communities and xenobiotic degrading microbial communities in freshwater river sediments. **Methods** The river water and sediments collected from the Wai-shuangh-si Stream in Taipei City, Taiwan were used to construct a model system in fish tanks to test the effects of parabens in laboratory. **Results** Tetracycline-, sulfamethoxazole- and paraben-resistant bacteria increased in all paraben treated river sediments. The order of the overall ability to produce an increment in sulfamethoxazole-, tetracycline- and paraben-resistant bacteria was MP > EP > PP > BP. The proportions of microbial communities associated with xenobiotic degradation also increased in all paraben-treated sediments. In contrast, penicillin-resistant bacteria in both the aerobic and anaerobic culture of paraben-treated sediments decreased drastically at the early stage of the experiments. The proportions of four microbial communities associated with the nitrogen cycle (anammox, nitrogen fixation, denitrification and dissimilatory nitrate reduction) and sulfur cycle (thiosulfate oxidation) largely increased after the 11th week in all paraben-treated sediments. Moreover, methanogens and methanotrophic bacteria increased in all paraben-treated sediments. In contrast, the nitrification, assimilatory sulfate reduction and sulfate-sulfur assimilation associated to microbial communities in the sediments were decreased by the parabens. The results of this study uncover the potential effects and consequences of parabens on microbial communities in a freshwater river environment.

## 1. Introduction

Parabens are the most prevalent additives in personal care products (PCPs) and cosmetics [1]. In 2006, parabens were used as preservatives in 22,000 types of cosmetics [2]. By 2018, the value of the global cosmetics market was at EUR 500 billion. The amounts of preservatives used in PCPs and cosmetics is expected to further increase in the coming years [3]. Methyl-, ethyl-, propyl-, and butyl-paraben (Me, EP, PP, and BP, respectively) are the most commonly used parabens [4].

Parabens are absorbed after dietary intake and dermal application. Parabens cause harmful effects on human health by disrupting the endocrine system [5]. Results of in vitro experiments have shown that parabens interfere with several hormone receptors, such as androgen-, estrogen-, progesterone-, glucocorticosteroid-, and peroxisome proliferator-activated receptors [4]. Long-term exposure to parabens has also been shown to increase breast cancer cell proliferation and migration [6].

The discharge of PCPs and cosmetics is the main source of parabens in wastewater treatment plants (WWTPs) and landfill leachate. Although WWTPs have a high removal efficiency for parabens, the levels of residual parabens are still high in WWTP effluents. According to Bledzka et al., the MP concentration in WWTP effluents in the U.S. was at 3830 ng/L [7]. The levels of MP and PP in urban streams in Japan were at 676 ng/L and 207 ng/L, respectively [8]. In European rivers, the highest levels of MP and PP were at 400 ng/L [9] and 69 ng/L [10], respectively. During the dry season, the highest values of MP and PP in the Xiangjiang River, China were at 3173.9 ng/L and 1040.4 ng/L, respectively [11].

Studies on the occurrence of parabens in sediments have shown that the highest concentrations observed for MP, EP, PP and BP were 476 ng/g, 60 ng/g, 64.5 ng/g, and 34 ng/g, respectively [12]. In China, the overall concentrations of parabens in the surface water of the Yellow River and the Huai River were 3.31–55.2 ng/L and 15.0–164 ng/L, respectively. The overall concentrations of parabens in the sediments of the Yellow River and the Huai River were 13.3–37.2 ng/g and 16.1–31.6 ng/g, respectively [13]. In sediment from Korean coastal waters, total concentrations of parabens ranged from 0.19 to 11.2 (mean: 2.40) ng/g dry weight [14].

MP, EP, PP and BP are biodegradable under aerobic conditions and partially degrade in anaerobic conditions. The biodegradability experiments of selected parabens in river water show half-lives ranging from 9.5 to 20 h [12]. In the study by Amin et al., *Pseudomonas beteli* and *Burkholderia latens* were found to degrade MP and PP [15]. Onuche et al. also reported the biodegradation of MP by five bacteria (*Klebsiella planticola*, *Vibro cholera*, *Pseudomonas beteli*, *Escherichia coli* and *Proteus vulgaris*) [16,17].

Parabens have become chemicals of emerging concern in aquatic environments. There are extensive studies regarding the occurrence, fate and behavior of parabens in aquatic environments [12,18]. However, little is known about the effects of parabens on microbial communities in river sediments. In this study, the effects of MP, EP, PP and BP on microbial communities of antimicrobial resistance, nitrogen/sulfur cycles and xenobiotic degradation in freshwater river sediments were investigated.

## 2. Materials and Methods

### 2.1. Chemicals

The chemicals methylparaben (MP), ethylparaben (EP), propylparaben (PP), butylparaben (BP), penicillin (pen), tetracycline (tet), and sulfamethoxazole (sul) were purchased from Sigma-Aldrich (Merck/Millipore Sigma, St. Louis, MO, USA). The structure and CAS number of the compounds used in this study are listed in Tables S1 and S2.

### 2.2. Experimental Design

The river water and sediments were collected from the Wai-shuangh-si Stream in Taipei City, Taiwan. The GPS coordinates of the sampling site are 25.07988, 121.49199. The setting of the fish tanks is shown in Figure S1A. River sediment with a volume of 10 cm × 45 cm × 45 cm and river water at 30 cm × 45 cm × 45 cm were placed in a 45 cm × 45 cm × 45 cm fish tank. A pump was used for water circulation. Five fish tanks for the control, MP, EP, PP and BP were set up (one paraben per tank). A total of 20 ppm of MP, EP, PP and BP were added into each tank every week. The timeline of the sediment sampling (for plate count and DNA extraction) is shown in Figure S1B.

### 2.3. Bacterial Culture and Plate Count

Agar plates composed of 1/3 tryptic soy broth (TSB) (Neogen Corporation, Lansing, MI, USA) and 1.5% agar (Neogen Corporation, Lansing, MI, USA) were used for the total plate count. As for the antibiotic- and paraben-resistant bacterial plate count, 1/3 TSB–agar plates with 100 μg/mL penicillin, 20 μg/mL tetracycline, 50 μg/mL sulfamethoxazole or 90 μg/mL of each paraben were used. These three antibiotics were chosen because they represent the three most commonly used antibiotic categories for human and animal health. The penicillin concentrations used in this study were based on the study of Alam et al. [19], and the tetracycline and sulfamethoxazole concentrations were based on the study of Choi et al. [20]. A total of 10 g of sediment and 20 mL of river water from the fish tanks were mixed and vortexed for 20 s. After standing for five minutes, the supernatant was used for the serial dilution and plate counting. The anaerobic conditions of bacterial cultures were achieved by using the BD GasPak™ EZ Anaerobe Gas Generating Pouch System (Becton, Dickinson and Company, Franklin Lakes, NJ, USA). The colonies grown on plates incubated under aerobic and anaerobic conditions under 25 °C for 3 days were subsequently counted.

### 2.4. Analysis of Chemical Compositions in Water

The water samples (50 mL for each sample) from the fish tanks were first filtered using a 1.20 µm filter and re-filtered with a 0.22 µm filter. The pH and ORP of water were analyzed using pH and ORP meters (METTLER TOLEDO, Greifensee, Switzerland). The levels of chemical oxygen demand (COD), sulfide (S^2−^), sulfate (SO_4_^2−^), ammonium (NH4^+^), nitrite (NO_2_^−^) and nitrate (NO_3_^−^) were determined using Merck test kits and the Spectroquant Nova 60 photometer (Merck KGaA, Darmstadt, Germany).

### 2.5. HPLC Analysis of Residual Parabens in Water

The water samples were collected and filtered using a 0.22 µm filter and subjected to HPLC analysis. The parabens were analyzed using an Agilent 1260 HPLC equipped with an InfinityLab PoroShell 120 EC-C18 column and monitored with a photodiode array detector at 254 nm (Agilent Technologies, Inc., Santa Clara, CA, USA). The solvents delivered by the analytical pump were acetonitrile (A) and water (5 mM KH_2_PO_4_) (B). The samples were eluted using 40/60 (A/B), with a flow rate of 1 mL/min. The recovery percentage was computed using the following formula: recovery percentage = (amount (concentration) of recovered preservative detected by HPLC/amount (concentration) of input preservative) × 100%. The recovery percentages for MP, EP, PP and BP were 96.2%, 95.3%, 95.6% and 94.3%, respectively. The detection limit for the parabens was 0.1 mg/L. All of the concentrations of MP, EP, PP and BP in the river water used for experiments were 0 ppm (under the detection limit of HPLC).

### 2.6. DNA Extraction, 16S Amplicon Preparation and NGS

DNA was extracted from the sediment samples using the PowerSoil DNA Isolation kit (QIAGEN, Venlo, The Netherlands). The V5–V8 variable regions of the 16S rRNA gene were amplified. The 5′ primer was composed of a 16S rRNA gene-specific sequence (5′-CCTACGGGNBGCASCAG-3′) and a sequencing adaptor (5′-TCGTCGGCAGCGTCAGATGTGTATAAGAGACAG3′). The 3′ primer was composed of sequencing adaptor (5′-GTCTCGTGGGCTCGGAGATGTGTATAAGAGACAG3′) and a 16S rRNA gene-specific sequence (5′-GACTACNVGGGTATCTAATCC-3′). The PCRs were performed using a 25 µL PCR mixture including a PCR buffer, 200 mM of each deoxynucleotide triphosphate, 10 pmol of each primer, 1.25 U of Taq polymerase, and 50 ng of template DNA. A reaction mixture without DNA was used as a negative control. The PCR procedure was as follows: 95 °C for 10 min, 30 cycles of 95 °C for 1 min, 55 °C for 1 min, 72 °C for 1 min, and a final step at 72 °C for 15 min. The PCR products were checked using 1.2% (*w/v*) agarose gel electrophoresis. The 16S amplicon sequencing was performed using the MiSeq platform (Illumina, Inc., San Diego, CA, USA) at the Cancer Progression Research Center, National Yang Ming Chiao Tung University, Taiwan.

### 2.7. Microbiome Data Analysis

The Trimmomatic software (v.0.35, http://www.usadellab.org/, accessed on 11 April 2023) was used for read trimming. The FLASH software (v.1.2.11, https://ccb.jhu.edu/software/FLASH/, accessed on 11 April 2023) was used to merge paired-end reads. The USEARCH software (v.11, http://www.drive5.com/usearch/, accessed on 11 April 2023) was used to remove chimeric sequences. Amplicon sequence variants (ASVs) were analyzed using DADA2. Diversity indexes were computed with the vegan package of R (v.4.1.3, https://www.r-project.org/, accessed on 11 April 2023). Taxonomic groups (phylum, class, order, family, genus) were assigned using the classifier (16s rRNA training set 18) in the Ribosomal Database Project (RDP Release 11, http://pyro.cme.msu.edu/, accessed on 11 April 2023). A similarity of 98% was used as the cutoff value for the sequence grouping (operational taxonomic units). Microbial genera with the nitrogen cycle, sulfur cycle, pathogenic bacteria and xenobiotic degradation pathways were retrieved from the Kyoto Encyclopedia of Genes and Genomes (KEGG) database [21] and combined with NGS data in this study. The microbial genera with significant different proportions in the sediment samples were identified using the Mann–Whitney U test. Nonmetric multidimensional scaling (NMDS) analysis was performed using the metaMDS function in the vegan package of R. The results of the NMDS analysis were plotted using the ggplot2 package of R.

## 3. Results

### 3.1. Increased Tetracycline-, Sulfamethoxazole- and Paraben-Resistant Microbes in Sediment

**Figure 1:**
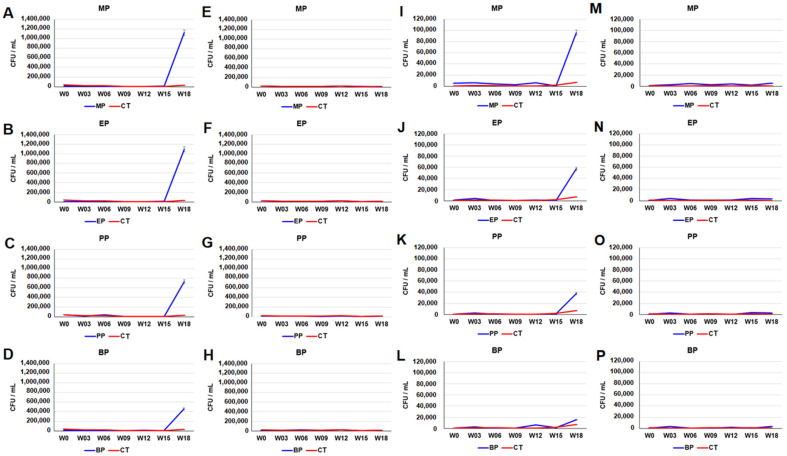
Plate counts of sulfamethoxazole- (**A**–**H**) and tetracycline- (**I**–**P**) resistant microbes in paraben-treated river sediments. (**A**–**D**,**I**–**L**) aerobic culture. (**E**–**H**,**M**–**P**) anaerobic culture. *Y*-axis indicates colony forming unit per mL (CFU/mL). *X*-axis indicates weeks (0–18th week). Data from triplicate assays are presented as the mean ± SE. MP: methylparaben, EP: ethylparaben, PP: propylparaben, BP: butylparaben, CT: control.

Bacterial culture and plate counting were used to examine the number of bacteria in paraben treated-sediments. As shown in Figure S2A–D, the plate counts of aerobic bacteria increased after the nineth week. The plate counts of anaerobic microbes increased after the 15th week (Figure S2E–H). The plate counts of sulfamethoxazole resistant bacteria in the aerobic cultures of paraben-treated sediments increased after the 15th week. The plate counts of sulfamethoxazole-resistant bacteria in the aerobic cultures of the MP-, EP-, PP- and BP-treated sediments on the 18th week were approximately 1.12 × 10^6^, 1.0 × 10^6^, 7.29 × 10^5^ and 4.56 × 10^5^ CFU/mL, respectively (Figure 1A–D). In contrast, the plate counts of sulfamethoxazole-resistant microbes in the anaerobic cultures of paraben-treated sediments showed a profile similar to that of the control sediments (Figure 1E–H). The plate counts of tetracycline-resistant bacteria in the aerobic cultures of paraben-treated sediments increased after the 15th week. The plate counts of tetracycline-resistant bacteria in the aerobic cultures of MP-, EP-, PP- and BP-treated sediments on the 18th week were approximately 9.62 × 10^4^, 5.74 × 10^4^, 3.74 × 10^4^ and 1.56 × 10^4^ CFU/mL, respectively (Figure 1I–L). The plate counts of tetracycline-resistant microbes in anaerobic cultures of paraben-treated sediments increased on the sixth week and after the 15th week. The plate counts of tetracycline-resistant bacteria in the anaerobic cultures of MP-, EP-, PP- and BP-treated sediments on the 18th week were approximately 5.1 × 10^3^, 3.53 × 10^3^, 2.34 × 10^3^, and 3.62 × 10^3^ CFU/mL, respectively (Figure 1M–P).

**Figure 2:**
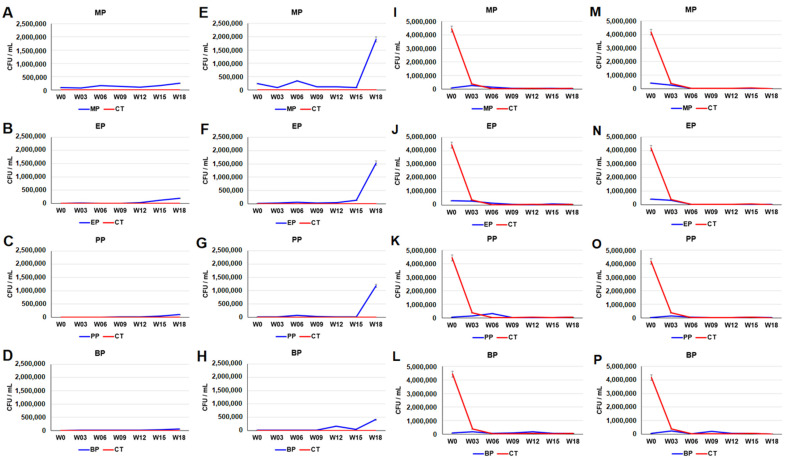
Plate counts of paraben- (**A**–**H**) and penicillin- (**I**–**P**) resistant microbes in paraben-treated river sediments. (**A**–**D**,**I**–**L**) aerobic culture. (**E**–**H**,**M**–**P**) anaerobic culture. *Y*-axis indicates colony forming unit per mL (CFU/mL). *X*-axis indicates weeks (0–18th week). Data from triplicate assays are presented as the mean ± SE. MP: methylparaben, EP: ethylparaben, PP: propylparaben, BP: butylparaben, CT: control.

The plate counts of paraben-resistant microbes in the anaerobic cultures of the paraben-treated sediments increased after the 15th week. The plate counts of the MP-, EP-, PP- and BP-resistant bacteria in the aerobic cultures of the MP-, EP-, PP- and BP-treated sediments on the 18th week were 2.5 × 10^5^, 1.93 × 10^5^, 1.07 × 10^5^, and 5.56 × 10^4^ CFU/mL, respectively (Figure 2A–D). The plate counts of the MP-, EP-, PP- and BP-resistant microbes in the anaerobic cultures of the MP-, EP-, PP- and BP-treated sediments on the 18th week were approximately 1.89 × 10^6^, 1.53 × 10^6^, 1.19 × 10^6^, and 4.06 × 10^5^ CFU/mL, respectively (Figure 2E–H). Compared together, the order of the overall ability to cause an increment of sulfamethoxazole-, tetracycline- and paraben-resistant microbes was MP > EP > PP > BP. In contrast, the plate counts of penicillin-resistant microbes in both the aerobic and anaerobic cultures of the paraben-treated sediments drastically decreased before the third week (Figure 2I–P).

### 3.2. Analysis of Chemical Compositions and Oxidation-Reduction Potential (ORP) in Water

The continuous addition of 20 ppm of paraben every week did not result in the accumulation of high levels of parabens in the water of the fish tanks (Figure S3). PP exhibited the lowest level among the four parabens. MP and EP exhibited very similar profiles.

**Figure 3:**
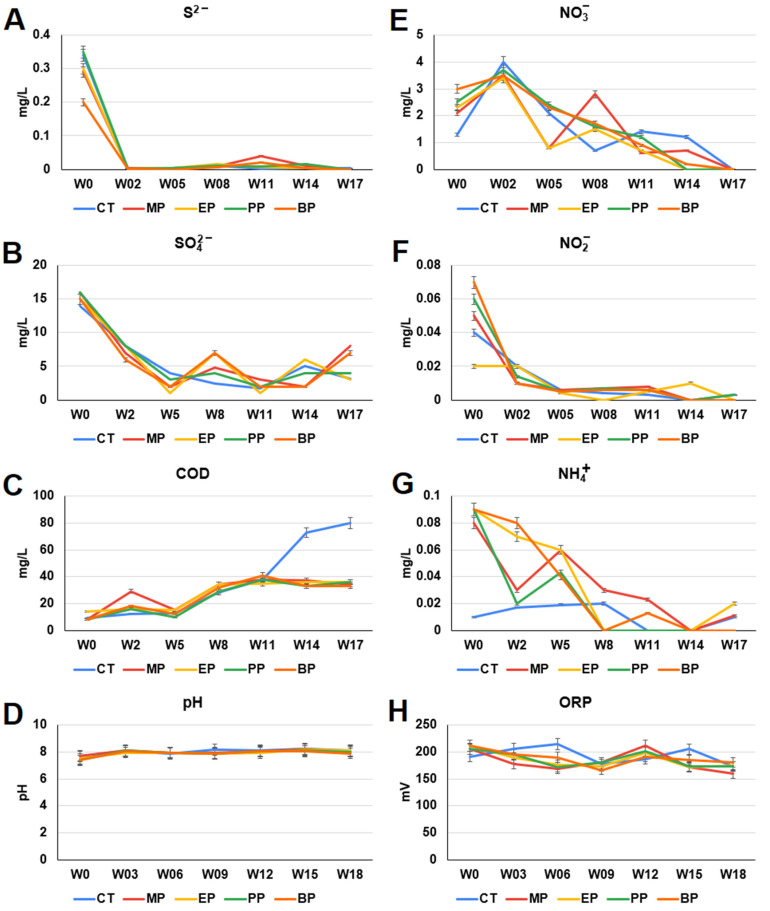
Chemical compositions of the river waters. (**A**) sulfide (S^2−^), (**B**) sulfate (SO_4_^2−^), (**C**) chemical oxygen demand (COD), (**D**) pH, (**E**) nitrate (NO_3_^−^), (**F**) nitrite (NO_2_^−^), (**G**) ammonium (NH_4_^+^), and (**H**) oxidation-reduction potential (ORP). *X*-axis indicates weeks (0–18th week). Data from triplicate assays are presented as the mean ± SE. MP: methylparaben, EP: ethylparaben, PP: propylparaben, BP: butylparaben, CT: control.

The chemical compositions and oxidation-reduction potential (ORP) of the control and paraben-treated river water were analyzed (Figure 3). The COD levels of the paraben-treated river water did not increase after the 11th week (Figure 3C). The ammonium (NH_4_^+^) levels of the paraben-treated river waters increased at the beginning of the experiments and then declined during the 18 weeks (Figure 3G). The sulfide (S^2−^), sulfate (SO_4_^2−^), ORP, pH, nitrate (NO_3_^−^) and nitrite (NO_2_^−^) profiles of the control and paraben-treated river waters were similar (Figure 3). It seems likely that the overall inorganic nitrogen (NH_4_^+^, NO_3_^−^ and NO_2_^−^) and sulfur (S^2−^ and SO_4_^2−^) compounds decreased by the end of the experiments.

### 3.3. Analysis of Microbial Community Compositions

**Figure 4:**
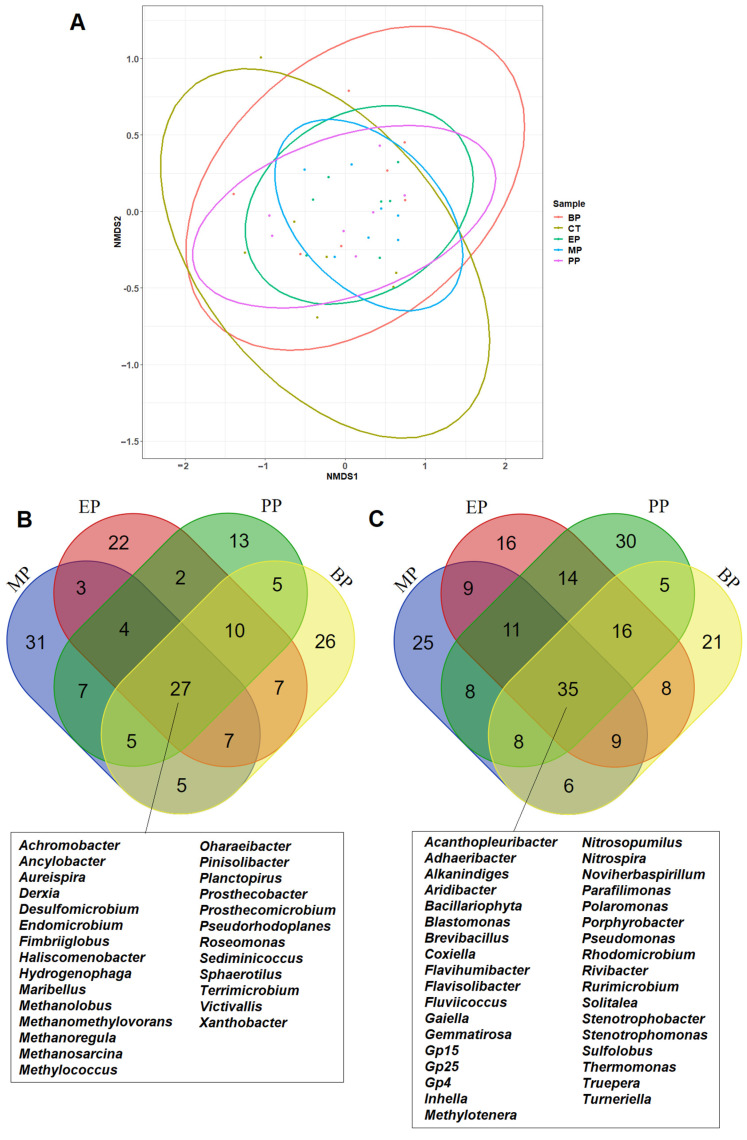
Identification of various common and known microbial genera from different paraben-treated river sediments. (**A**) Comparison (NMDS analysis) of microbiome compositions between different paraben-treated river sediments. (**B**) Venn diagram analysis and number of microbial genera increased in paraben treated river sediments. (**C**) Venn diagram analysis and number of microbial genera decreased in paraben treated river sediments. MP: methylparaben, EP: ethylparaben, PP: propylparaben, BP: butylparaben.

The 16S amplicon sequencing was used to analyze the microbial community compositions of the control and paraben-treated sediments. The overall proportions of known microbial communities at the phylum- and genus-level in the control and paraben-treated sediments are shown in Figures S4 and S5. Proteobacteria was the major phylum (45–95%) for all samples (Figure S4). The genus *Methylotenera* exhibited the highest proportion in the beginning of the experiments, but declined after the eighth week in the control and paraben-treated sediments (Figure S5). The results of the NMDS analysis are shown in Figure 4A. Overlapping ellipses indicate the presence of a core microbiome composition in sediments after the paraben treatments. The largest diameters of these ellipses have the following order: CT > BP > PP > EP = MP, which may indicate that the proportional variations in microbiome composition in the sediments decreased after the paraben treatments. This observation is consistent with the order of the plate counts of tetracycline-, sulfamethoxazole- and paraben-resistant microbes in the paraben-treated sediments (MP > EP > PP > BP). The proportions of twenty-seven microbial genera (including four methanogenic archaea: *Methanolobus*, *Methanoregula*, *Methanomethylovorans* and *Methanosarcina*) increased in all the paraben-treated river sediments (Figure 4B). The proportions of thirty-five microbial genera decreased in all the paraben-treated river sediments (Figure 4C).

### 3.4. Microbial Community Associated with the Nitrogen Cycle

**Figure 5:**
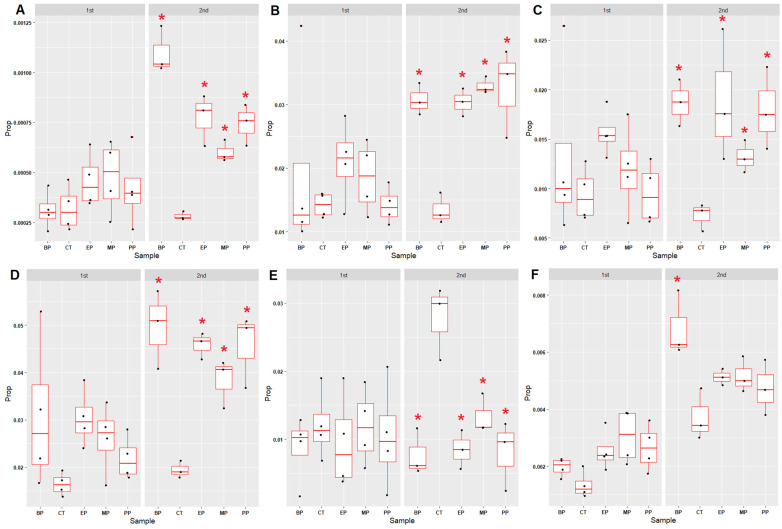
Proportion changes in the nitrogen cycle-associated microbial communities in the paraben-treated river sediments. (**A**) Anammox (anaerobic ammonium oxidation) (**B**) Nitrogen fixation (M00175: nitrogen => ammonia) (**C**) Denitrification (M00529: nitrate => nitrogen) (**D**) Dissimilatory nitrate reduction (M00530: nitrate => ammonia) (**E**) Nitrification (M00528: ammonia => nitrite) (**F**) Assimilatory nitrate reduction (M00531: nitrate => ammonia). “1st” indicates the meaning of the period between week 0 and week 8. “2nd” indicates the meaning of the period between week 8 and week 17. Red star indicates the *p* value of the Mann–Whitney U test < 0.05 (compared with control (CT)). Prop: proportions of microbial genera. “M00xxx” indicates the meaning of the KEGG module ID number. MP: methylparaben, EP: ethylparaben, PP: propylparaben, BP: butylparaben, CT: control.

To uncover the effects of parabens on the nitrogen cycle in sediments, six nitrogen cycle-associated microbial groups (anaerobic ammonium oxidation (anammox), nitrogen fixation, nitrification, denitrification, dissimilatory nitrate reduction and assimilatory nitrate reduction) were examined (Figures S6–S11). The proportions of four nitrogen cycle-associated microbial groups (anammox (Figure 5A and Figure S6), nitrogen fixation (Figure 5B and Figure S7), denitrification (Figure 5C and Figure S8) and dissimilatory nitrate reduction (Figure 5D and Figure S9) increased in all the paraben-treated sediments after the eighth week. In contrast, the proportions of the nitrification-associated microbial communities in the paraben-treated sediments decreased (Figure 5E and Figure S10). This result suggests that nitrification might be inhibited by parabens in all the paraben-treated sediments after the eighth week. Only BP led to an increase of assimilatory nitrate reduction-associated microbial communities after the eighth week (Figure 5F and Figure S11).

### 3.5. Microbial Community Associated with the Sulfur Cycle

**Figure 6:**
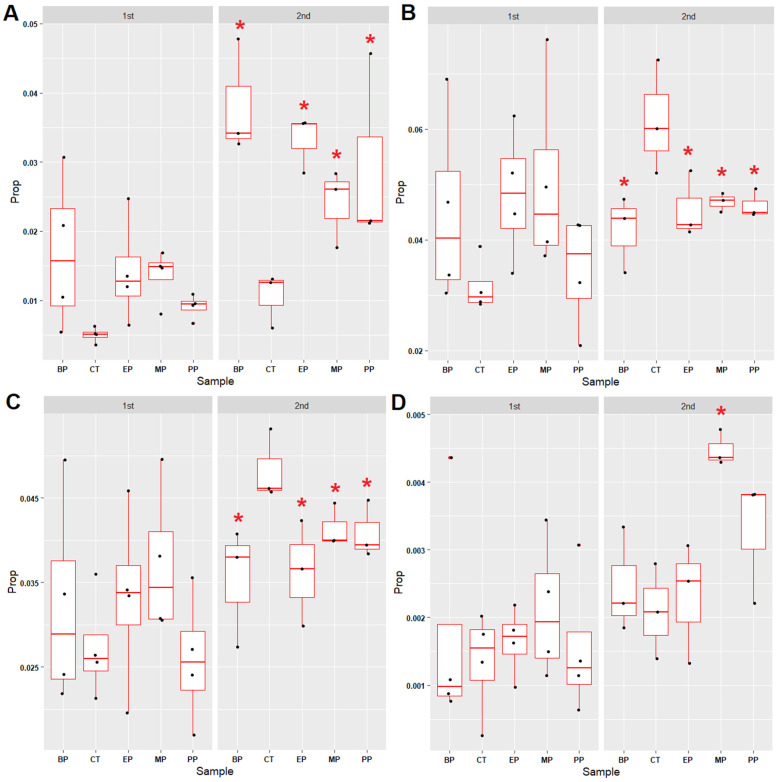
Proportion changes of S cycle-associated microbial communities in the paraben-treated river sediments. (**A**) Thiosulfate oxidation (M00595: thiosulfate => sulfate). (**B**) Assimilatory sulfate reduction (M00176: sulfate => H_2_S). (**C**) Sulfate-sulfur assimilation (M00616). (**D**) Dissimilatory sulfate reduction (M00596: sulfate => H_2_S). “1st” indicates the meaning of the period between week 0 and week 8. “2nd” indicates the meaning of the period between week 8 and week 17. Red star indicates the *p* value of the Mann–Whitney U test < 0.05 (compared with control (CT)). Prop: proportions of microbial genera. “M00xxx” indicates the meaning of the KEGG module ID number. MP: methylparaben, EP: ethylparaben, PP: propylparaben, BP: butylparaben, CT: control.

To uncover the effects of parabens on sulfur metabolism in sediments, four sulfur cycle-associated microbial groups (assimilatory sulfate reduction, dissimilatory sulfate reduction, thiosulfate oxidation and sulfate-sulfur assimilation) were examined (Figures S12–S15). The proportions of thiosulfate oxidation microbial communities increased after the eighth week in all the paraben-treated sediments (Figure 6A and Figure S12). In contrast, the proportions of the microbial communities associated with assimilatory sulfate reduction (Figure 6B and Figure S13) and sulfate-sulfur assimilation (Figure 6C and Figure S14) in the paraben-treated sediments decreased. These results indicate that the assimilatory sulfate reduction and sulfate-sulfur assimilation might be inhibited by parabens. Only MP led to an increase of microbial communities associated with dissimilatory sulfate reduction after the eighth week (Figure 6D and Figure S15).

### 3.6. Microbial Communities Associated with Xenobiotics Degradation and Pathogenic Bacteria

**Figure 7:**
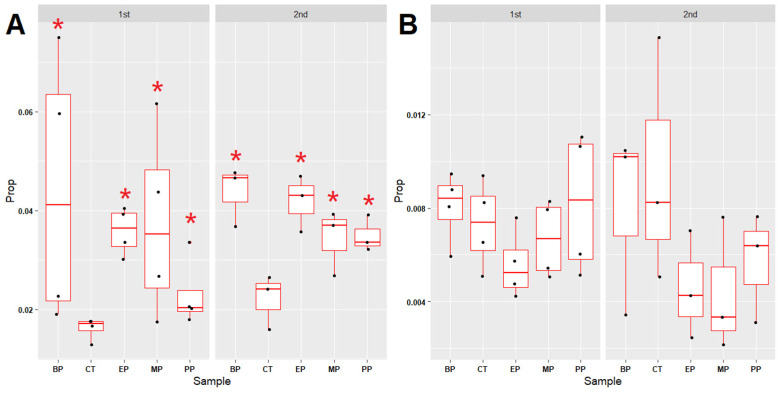
Proportion changes of microbial communities in the paraben-treated river sediments. (**A**) Bacterial genera associated with xenobiotics degradation. (**B**) Microbial genera with potential pathogenic bacteria. “1st” indicates the meaning of the period between week 0 and week 8. “2nd” indicates the meaning of the period between week 8 and week 17. Red star indicates the *p* value of the Mann–Whitney U test < 0.05 (compared with control (CT)). Prop: proportions of microbial genera. “M00xxx” indicates the meaning of the KEGG module ID number. MP: methylparaben, EP: ethylparaben, PP: propylparaben, BP: butylparaben, CT: control.

**Figure 8:**
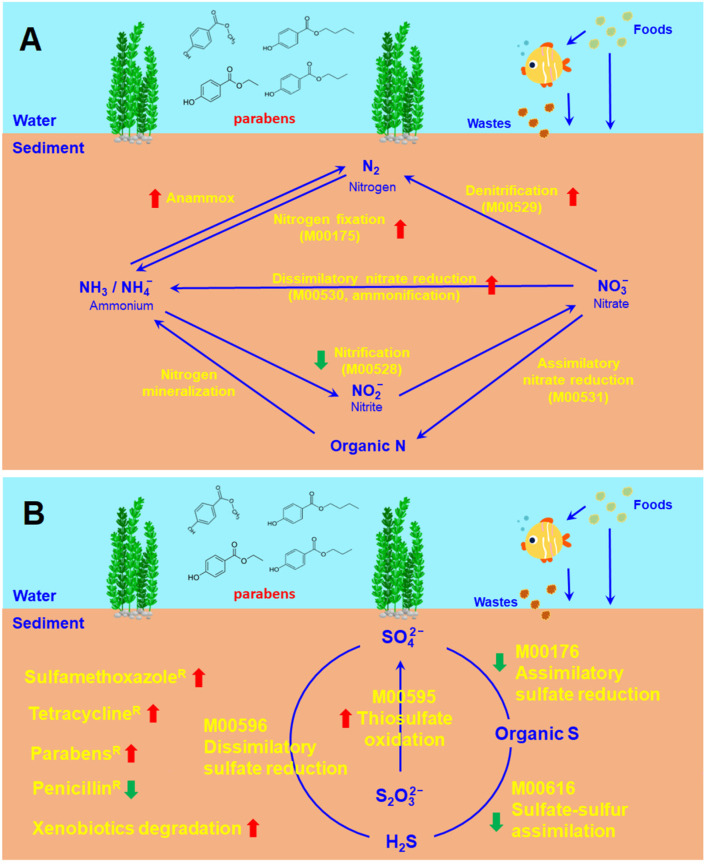
Effects of parabens on microbiomes in the freshwater river sediments revealed in this study. (**A**) Nitrogen cycle-associated microbial communities. (**B**) Sulfur cycle-associated microbial communities. Red arrows indicate increase of microbes. Green arrows indicate decrease of microbes. “M00xxx” indicates KEGG module ID number.

The proportions of the microbial communities associated with xenobiotic degradation increased in all the paraben-treated sediments (Figure 7A and Figure S16). In contrast, the proportions of the microbial communities associated with potential pathogenic bacteria did not exhibit differences in control or in all the paraben-treated sediments (Figure 7B and Figure S17). Changes in the microbial communities in the river sediments caused by parabens are summarized in Figure 8.

## 4. Discussion

Most of the antibiotic- and paraben-resistant bacteria in the paraben-treated sediments increased after the 15th week, which suggests that the continuous addition of parabens may lead to adaptation/selection pressure on the microbiome in sediments. Moreover, the continuous addition of 20 ppm of parabens every week did not result in the accumulation of high levels of parabens in the water of the fish tanks (Figure S3). The proportions of xenobiotic degradation-associated microbial communities increased in all the paraben-treated sediments (Figure 7A and Figure S16). This observation provides an explanation for the increment in sulfonamide-, tetracycline- and paraben-resistant bacteria and the degradation of continuously added parabens in the water of the fish tanks.

The profile of the decrease in penicillin-resistant microbes was not consistent with the profiles of the increment in tetracycline-, sulfamethoxazole- and paraben-resistant and xenobiotic-degrading microbes. This might be due to collateral sensitivity (CS). CS is a situation where resistance to one drug confers increased susceptibility to another drug (for example, preservatives, antibiotics or anti-cancer drugs). CS typically means that the inhibition of (bacterium or cell) growth can be achieved with lower concentrations of a drug. For preservatives or antibiotics, CS means the faster and stronger inhibition or killing of the resistant bacterium [22]. CS is a promising approach to counteract the rising problem of antibiotic resistance (ABR). Uncovering the antibiotic resistome provides new opportunities for therapeutic intervention [22,23]. The associations between CS and the evolution of β-lactamase genes have been reported [24,25]. Moreover, it has been found that collateral sensitivity is associated with antibiotic-resistant plasmids that carry β-lactamase genes [26]. Most penicillin resistance is due to the horizontal gene transfer of the penicillin-resistant gene (penicillinase) between bacteria [27]. Therefore, whether parabens can reduce/inhibit horizontal gene transfer between bacteria in sediments is worth further study.

It is very interesting that all paraben treatments led to the convergence of microbiome composition in the sediments (Figure 4A). It seems likely that parabens applied selection pressure to shape a more stable microbiome composition in the paraben-treated sediments (Figure 4). In addition, all paraben treatments led to an increase in the amount of four methanogens in the sediments (Figure 4B). The overall effects of the four parabens on the microbial communities in the river sediments were similar. This may be due to the similarity of the molecular structures of the four parabens. Moreover, the degradation intermediates of the four parabens in the sediments may also be very similar. It has been shown that p-hydroxybenzoic acid (p-HBA) is the common metabolite of MP, EP, PP and BP and is readily biodegradable under aerobic conditions [28]. Parabens can be degraded into phenol and p-HBA and can be used as carbon sources by microorganisms [29,30]. Under aerobic conditions, the degradation pathway of parabens has two steps: firstly, the hydrolysis of the ester bond to produce p-HBA, followed by a decarboxylation step to produce phenol [31].

All of the four parabens exhibited great effects on the nitrogen and sulfur cycle-associated microbial communities in the sediments. The addition of parabens led to a decreased ORP (Figure 3H), which is an indicator of decreased water quality. Moreover, the ammonium levels of the paraben-treated river water were higher than those of the control river water before the fifth–eighth week (Figure 3G). A high ammonium level is one of the major environmental pollutants in freshwater aquatic systems that is physiologically harmful to aquatic organisms [31]. Moreover, a high ammonium level stimulates the growth of cyanobacteria blooms, and represents a potential hazard to human health [32,33].

Freshwater aquatic systems are hotspots of nitrogen cycling processes. The growing intensification of anthropogenic activities leads to the great amount of nutrients added to freshwater aquatic ecosystems [34]. The nitrate (NO_3_^-^) delivered to aquatic systems is consumed by benthic microbes using two processes: denitrification [35] and anammox (anaerobic ammonium oxidation) [36]. Dissimilatory nitrate reduction to ammonium retains nitrogen within aquatic ecosystems [37]. These NO_3_^−^ competition processes are mediated by specific prokaryotes with abilities to change the geochemical conditions of the benthic environments [38]. Geochemical controls on dissimilatory nitrate reduction, denitrification and anammox have been revealed in a number of freshwater aquatic ecosystems. Dissimilatory nitrate reduction is favored over denitrification in aquatic ecosystems with high ratios of organic carbon (OC) to NO_3_^−^ [38,39] and high levels of sulfate reduction and sulfide oxidation [40]. In this study, the levels of sulfide (S^2−^), sulfate (SO_4_^2−^), nitrite (NO_2_^−^), nitrate (NO_3_^−^) and ammonium (NH_4_^+^) decreased in the river water in all settings (Figure 3), which may reflect the incorporation of inorganic elements into organic compounds (for example, assimilatory nitrate reduction, assimilatory sulfate reduction, sulfate-sulfur assimilation and growth of microbes). It has been found that the nitrogen cycle can be coupled with the sulfur cycle [41]. A novel microbe-mediated process (sulfammox), using SO_4_^2−^ as an electron acceptor, coupling NH_4_^+^ oxidation with SO_4_^2−^ reduction to form N_2_ under anaerobic conditions, has been identified in natural environments [42]. The sulfammox process provides a connection between the N and S cycles, and may promote N_2_ release in natural environments [43]. Therefore, another reason for the decrease of nitrite (NO_2_^−^), nitrate (NO_3_^−^), ammonium (NH_4_^+^), sulfide (S^2−^) and sulfate (SO_4_^2−^) in the paraben-treated river waters may also be due to the increase in N and S cycle coupling which leads to the release of nitrogen from water.

A study by Dang et al. found that antibiotics stress led to an increase of the genes associated with the nitrate reduction, denitrification and dissimilatory nitrogen transformation pathways in water samples from the Danjiangkou Reservoir in China [44]. ^15^N-labelling analysis revealed that the denitrification was the major pathway for nitrogen removal (approximately 57.1% of nitrogen loss). The results of this study indicate that preservatives may exhibit similar effects (as antibiotics) on nitrogen transformation pathways in freshwater rivers.

## 5. Conclusions

The results of this study indicate that parabens have great effects on microbial community compositions in freshwater river sediments. As a consequence, changes in microbial communities led to the increase in tetracycline-, sulfamethoxazole- and paraben-resistant microbes and xenobiotic-degrading microbes. Moreover, parabens cause changes in the microbial communities associated with the nitrogen and sulfur cycles in freshwater river sediments. The increase of methanogens in sediments may affect methane production in the freshwater environment. Combined, parabens may lead to changes in the chemical element distribution (efficiencies of assimilation and dissimilation) of the organic and inorganic parts of aquatic environments. The effects of parabens on the increase in tetracycline-, sulfamethoxazole- and paraben-resistant microbes in sediments have the following order: MP > EP > PP > BP. In contrast, the overall effects of parabens on the changes in chemical element distribution are similar. The effects of parabens on the nitrogen budget, sulfur cycle and methane production in freshwater aquatic environments are worthy of more in-depth investigations.

## Data Availability

The data presented in this study are available in Supplementary Materials.

## Supplementary Materials

The following supporting information can be downloaded at: https://www.mdpi.com/article/10.3390/toxics11040387/s1, Figure S1: Experimental designs, Figure S2: Total plate counts of bacteria in paraben treated river sediments, Figure S3: Residual parabens in each fish tank, Figure S4: Microbial communities (phylum level) in the paraben treated river sediments, Figure S5: Microbial communities (genus level) in the paraben treated river sediments, Figure S6: Anammox (anaerobic ammonium oxidation) associated microbial communities in the paraben treated river sediments, Figure S7: Nitrogen fixation (M00175: nitrogen => ammonia) associated microbial communities in the paraben treated river sediments, Figure S8: Denitrification (M00529: nitrate => nitrogen) associated microbial communities in the paraben treated river sediments, Figure S9: Dissimilatory nitrate reduction (M00530: nitrate => ammonia) associated microbial communities in the paraben treated river sediments, Figure S10: Nitrification (M00528: ammonia => nitrite) associated microbial communities in the paraben treated river sediments, Figure S11: Assimilatory nitrate reduction (M00531: nitrate => ammonia) associated microbial communities in the paraben treated river sediments, Figure S12: Thiosulfate oxidation (M00595: thiosulfate => sulfate) associated microbial communities in the paraben treated river sediments, Figure S13: Assimilatory sulfate reduction (M00176: sulfate => H_2_S) associated microbial communities in the paraben treated river sediments, Figure S14: Sulfate-sulfur assimilation (M00616) associated microbial communities in the paraben treated river sediments, Figure S15: Dissimilatory sulfate reduction (M00596: sulfate => H_2_S) associated microbial communities in the paraben treated river sediments, Figure S16: Microbial genera associated with xenobiotics degradation bacteria in the paraben treated river sediments, Figure S17: Proportions of microbial genera with potential pathogenic bacteria in the paraben treated river sediments, Table S1: Target compounds used in this study, Table S2. Antibiotics used in this study, EXCLE S1: Microbial communities.
