# Supplementary material for: Parabens Increase Sulfamethoxazole-, Tetracycline- and Paraben-Resistant Bacteria and Reshape the Nitrogen/Sulfur Cycle-Associated Microbial Communities in Freshwater River Sediments
Source: Toxics. 2023 Apr 18;11(4):387. doi: 10.3390/toxics11040387 (PMC10142436; doi:10.3390/toxics11040387)
Supplement: Supplementary file 1 [file toxics-11-00387-s001.zip › SFigures_STables.pdf]

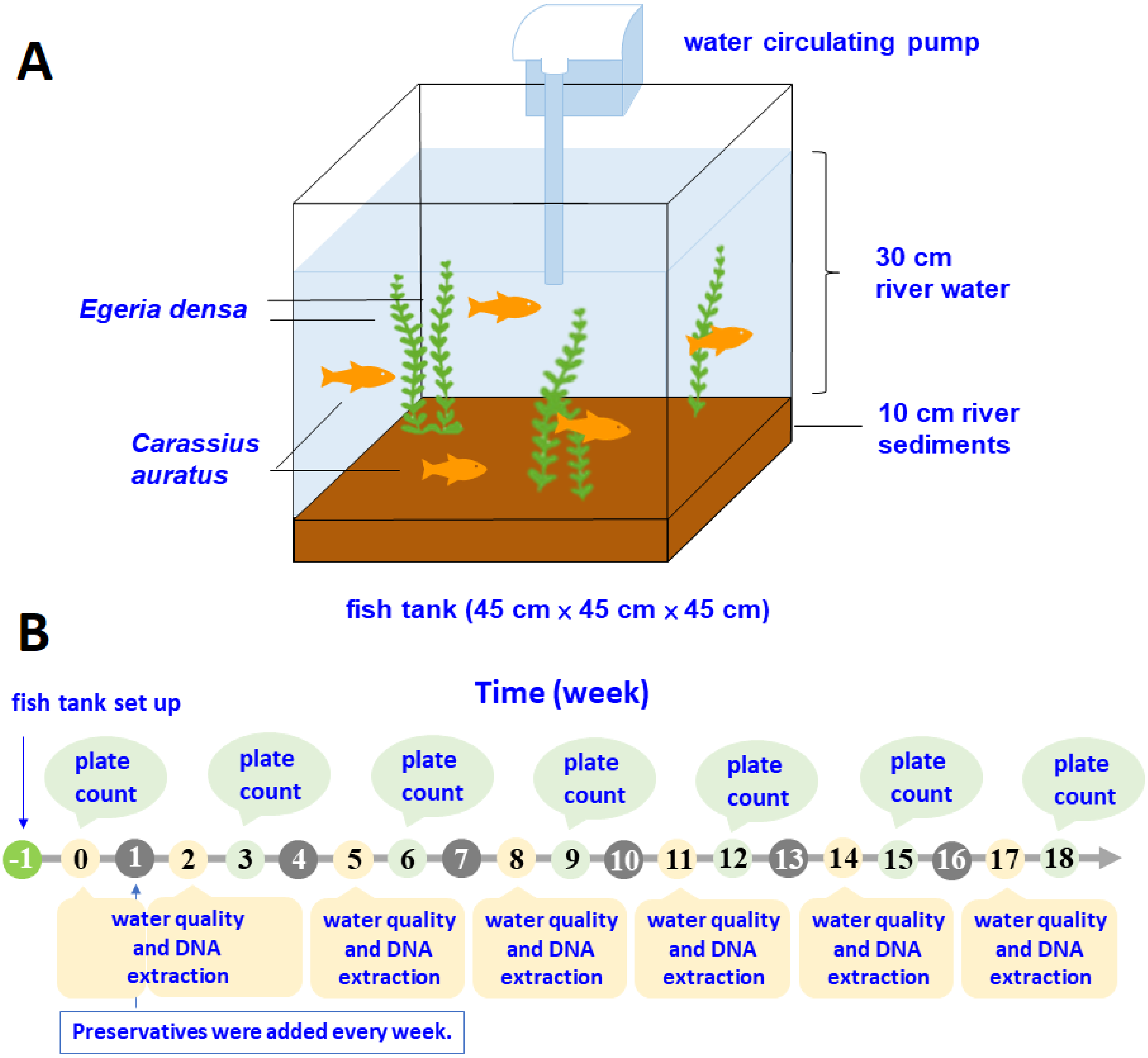

Figure S1. Experimental designs. (A) Compositions of fish tank. (B) Timeline of sampling and experiments in this study.

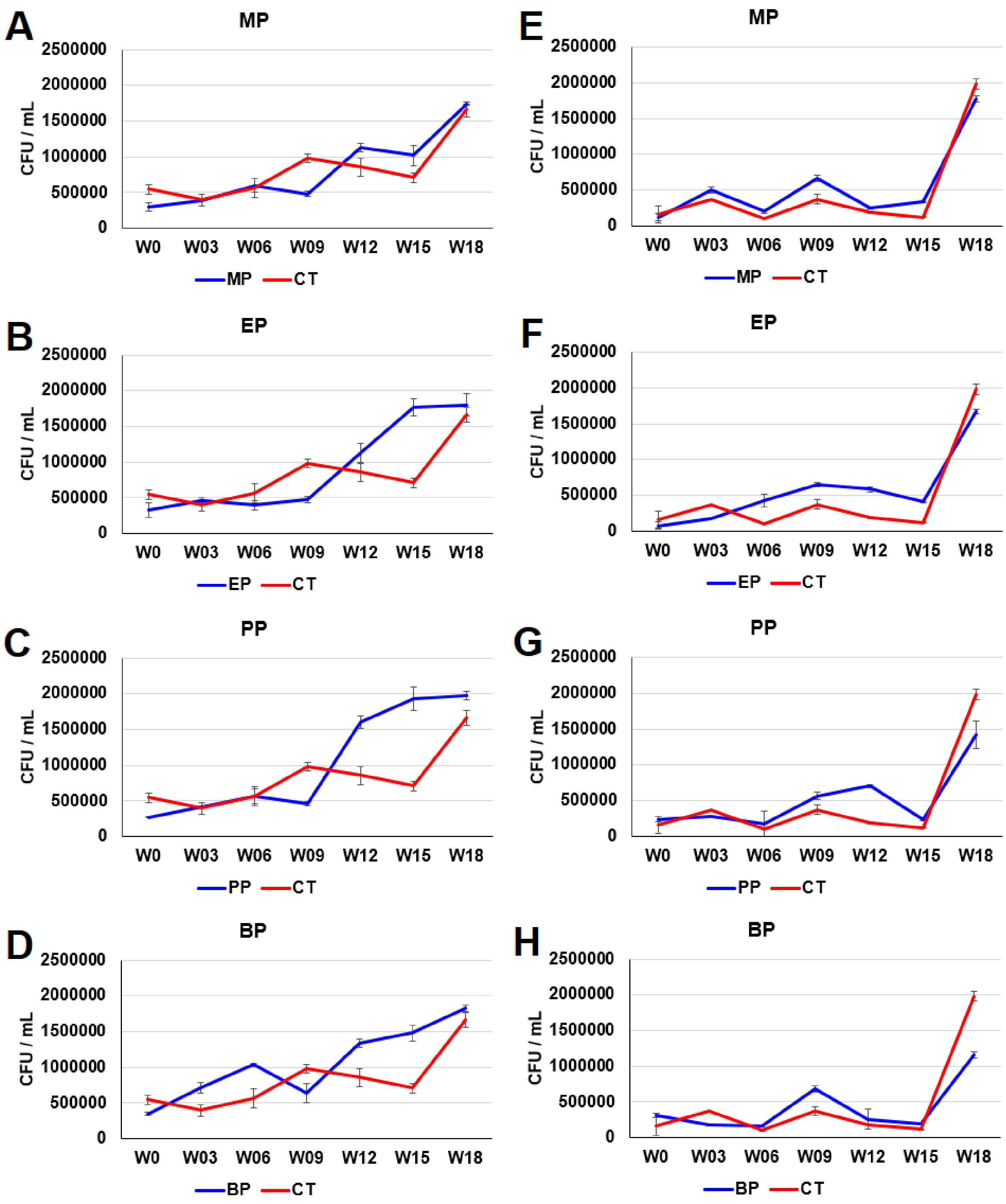

Figure S2. Total plate counts of bacteria in paraben treated river sediments. A-D: aerobic culture. E-H: anaerobic culture. Y-axis indicates colony forming unit per mL (CFU/mL). X-axis indicates weeks (0-18th week). Data from triplicate assays are presented as the mean ± SE. MP: methylparaben, EP: ethylparaben, PP: propylparaben, BP: butylparaben, CT: control.

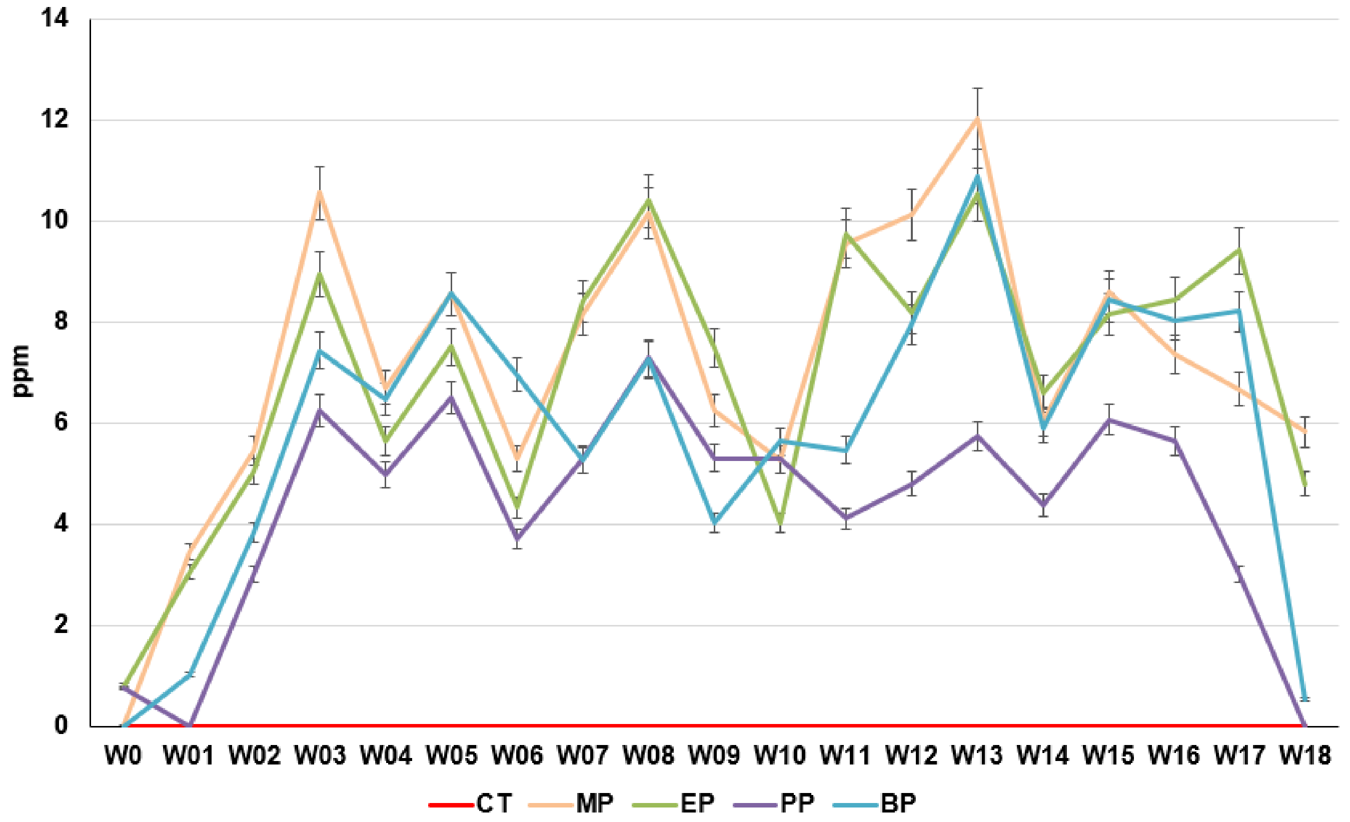

Figure S3. Residual parabens in each fish tank. Y-axis indicates residual paraben (parts per million, ppm). X-axis indicates weeks (0-18th week). Data from triplicate assays are presented as the mean ± SE. MP: methylparaben, EP: ethylparaben, PP: propylparaben, BP: butylparaben, CT: control.

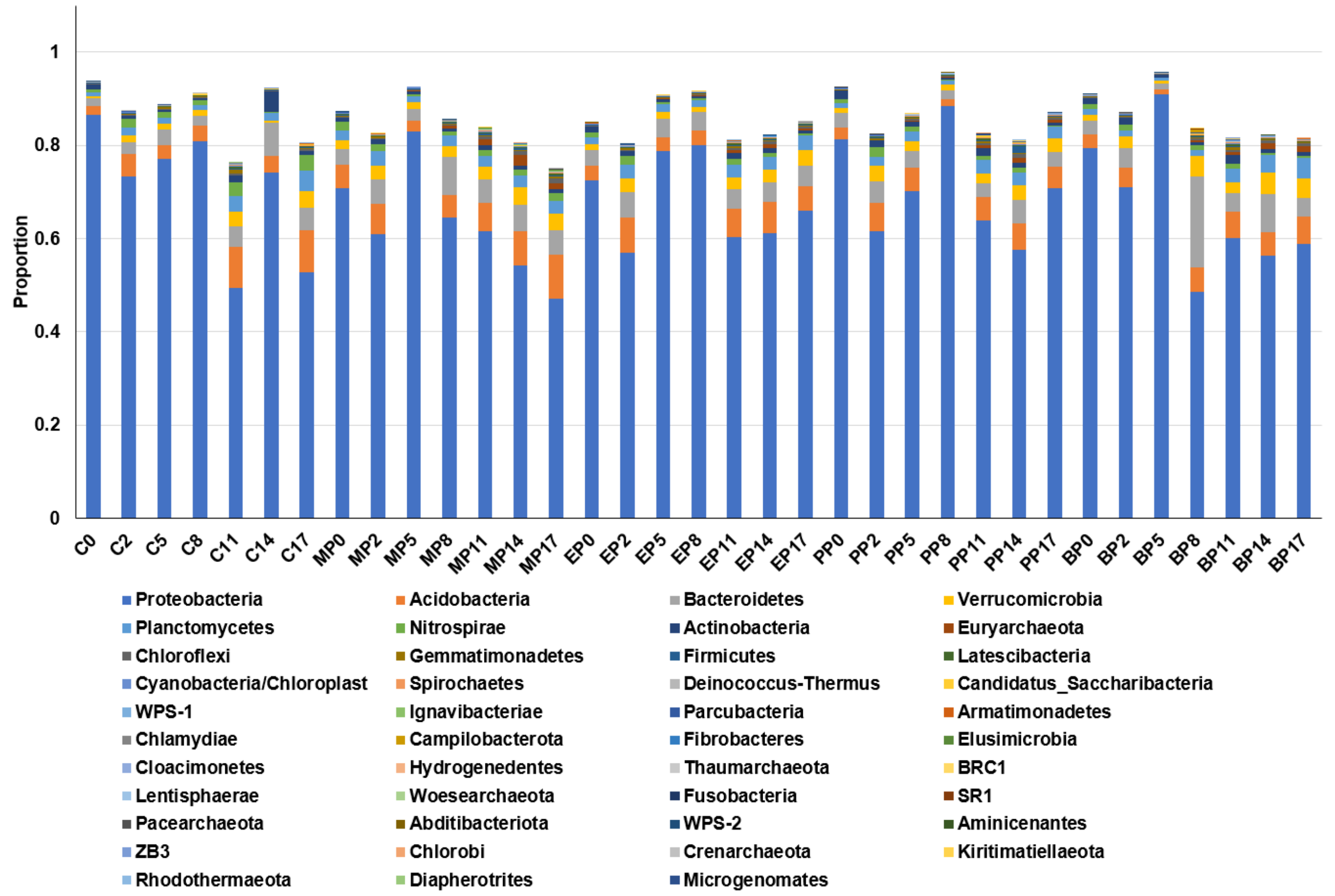

Figure S4. Microbial communities (phylum level) in the paraben treated river sediments. MP: methylparaben, EP: ethylparaben, PP: propylparaben, BP: butylparaben, C: control.

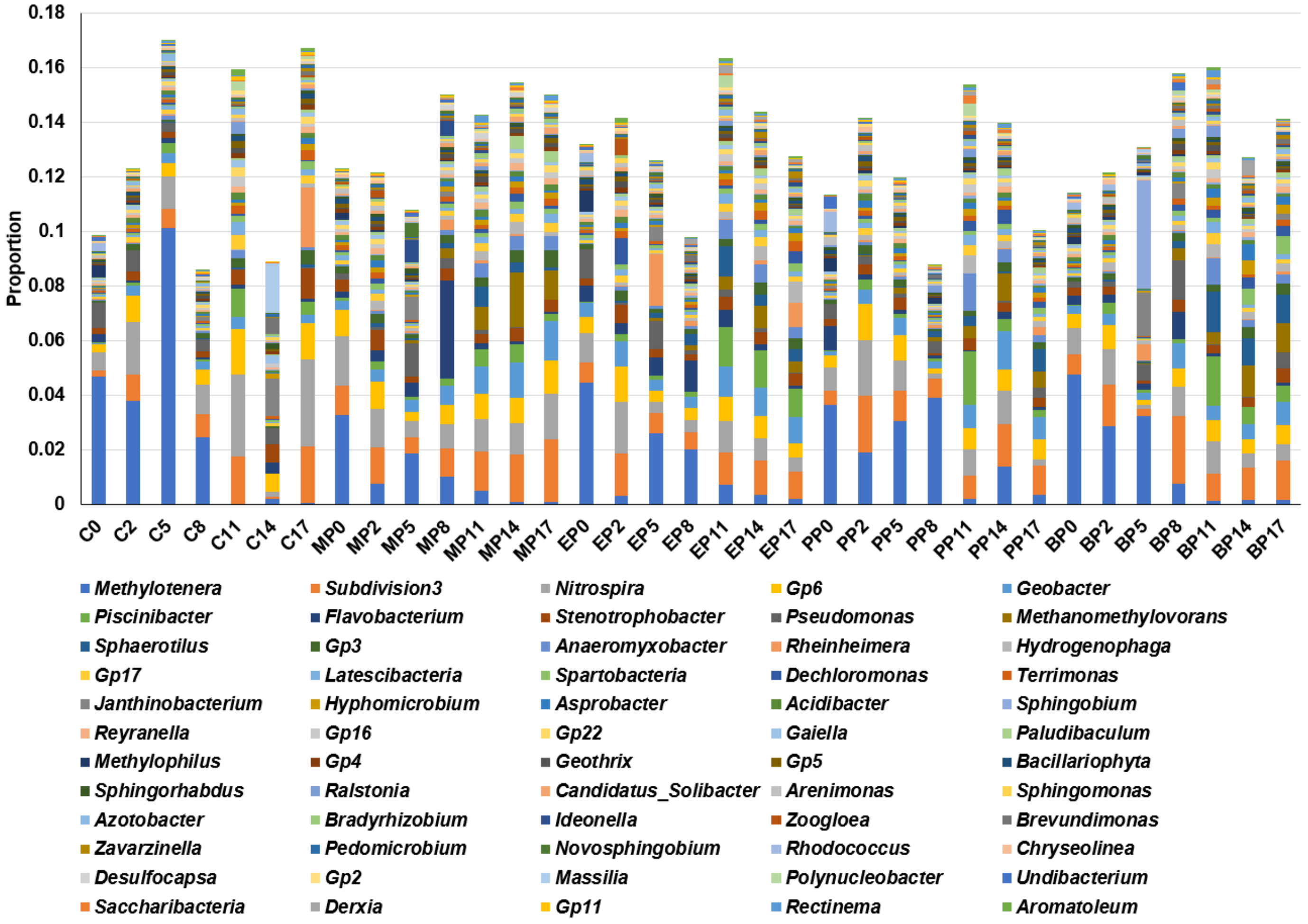

Figure S5. Microbial communities (genus level) in the paraben treated river sediments. MP: methylparaben, EP: ethylparaben, PP: propylparaben, BP: butylparaben, C: control.

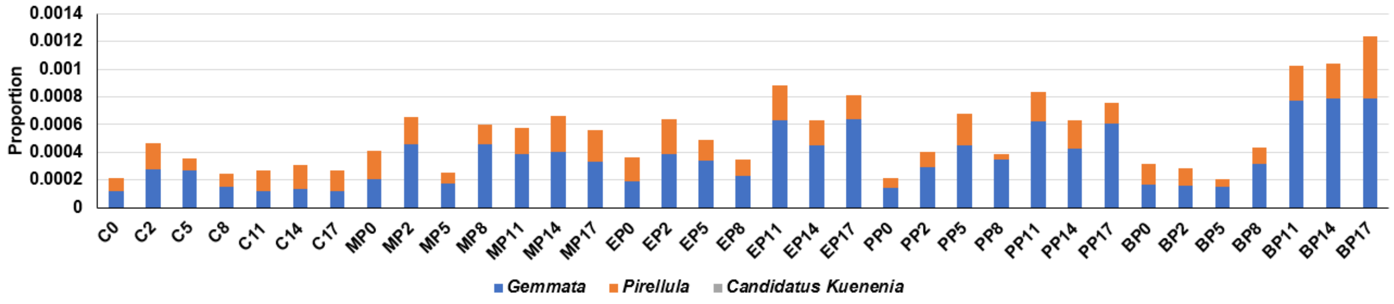

Figure S6. Anammox (anaerobic ammonium oxidation) associated microbial communities in the paraben treated river sediments. MP: methylparaben, EP: ethylparaben, PP: propylparaben, BP: butylparaben, C: control.

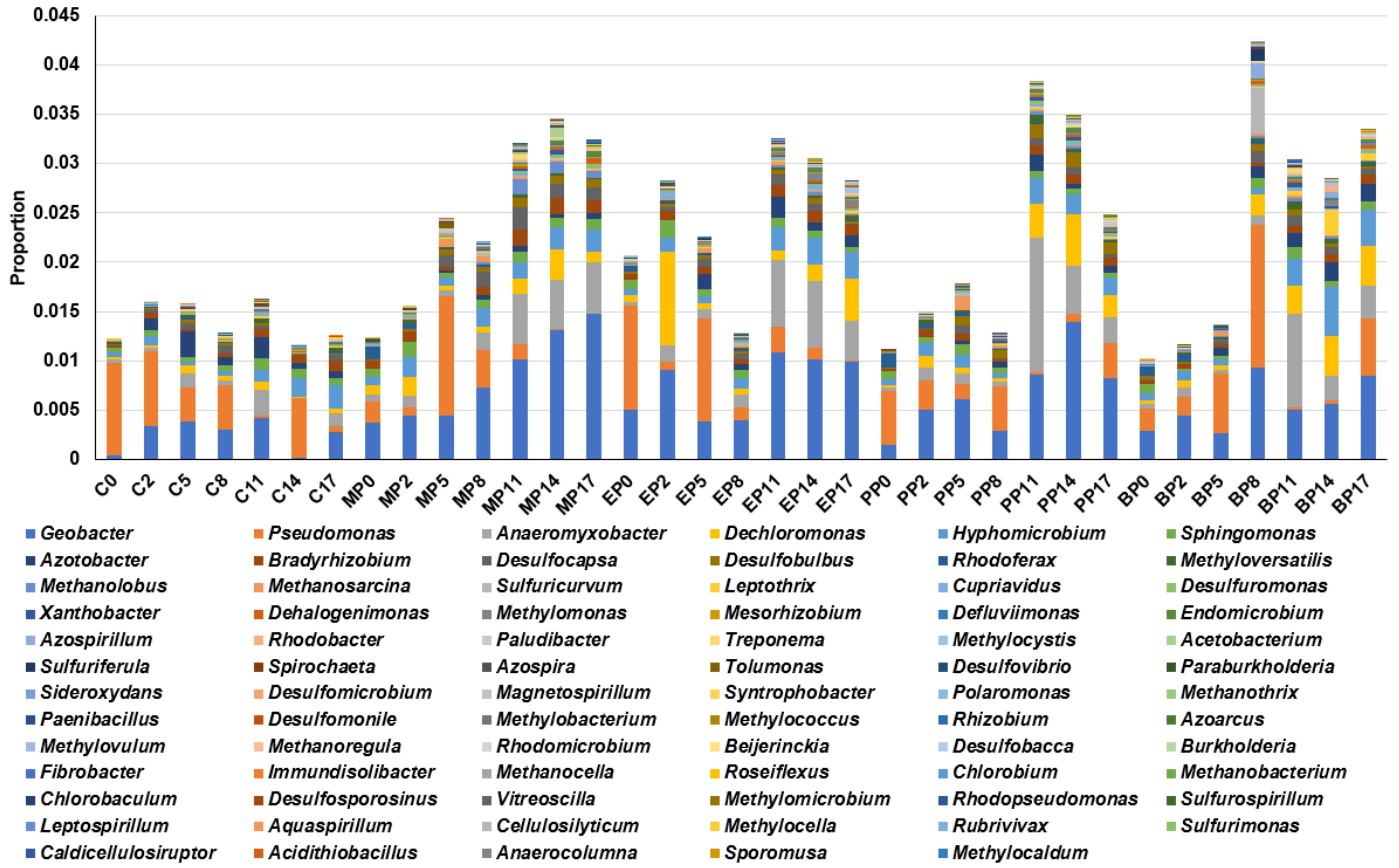

Figure S7. Nitrogen fixation (M00175: nitrogen => ammonia) associated microbial communities in the paraben treated river sediments. MP: methylparaben, EP: ethylparaben, PP: propylparaben, BP: butylparaben, C: control.

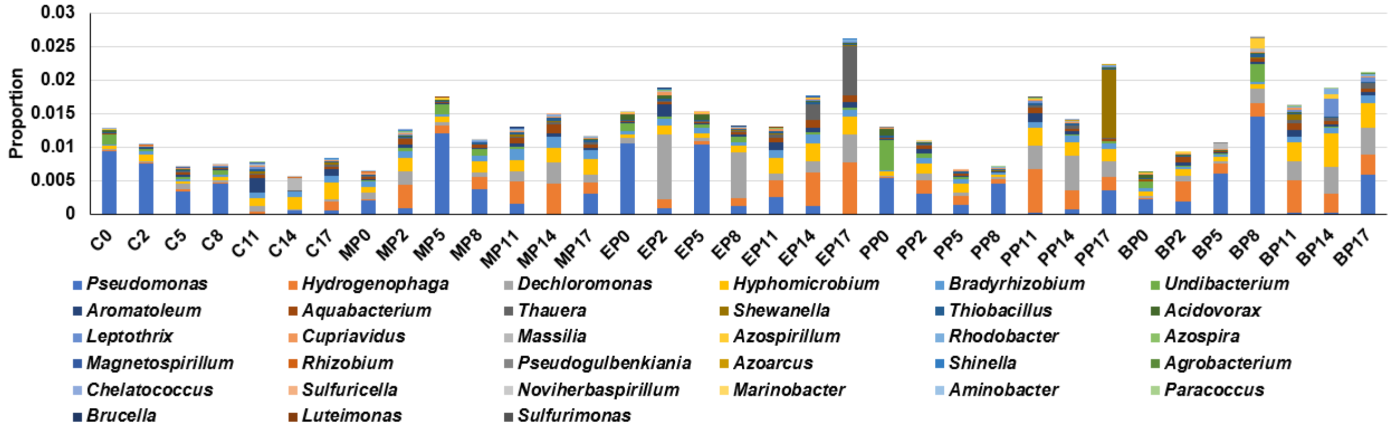

Figure S8. Denitrification (M00529: nitrate => nitrogen) associated microbial communities in the paraben treated river sediments. MP: methylparaben, EP: ethylparaben, PP: propylparaben, BP: butylparaben, C: control.

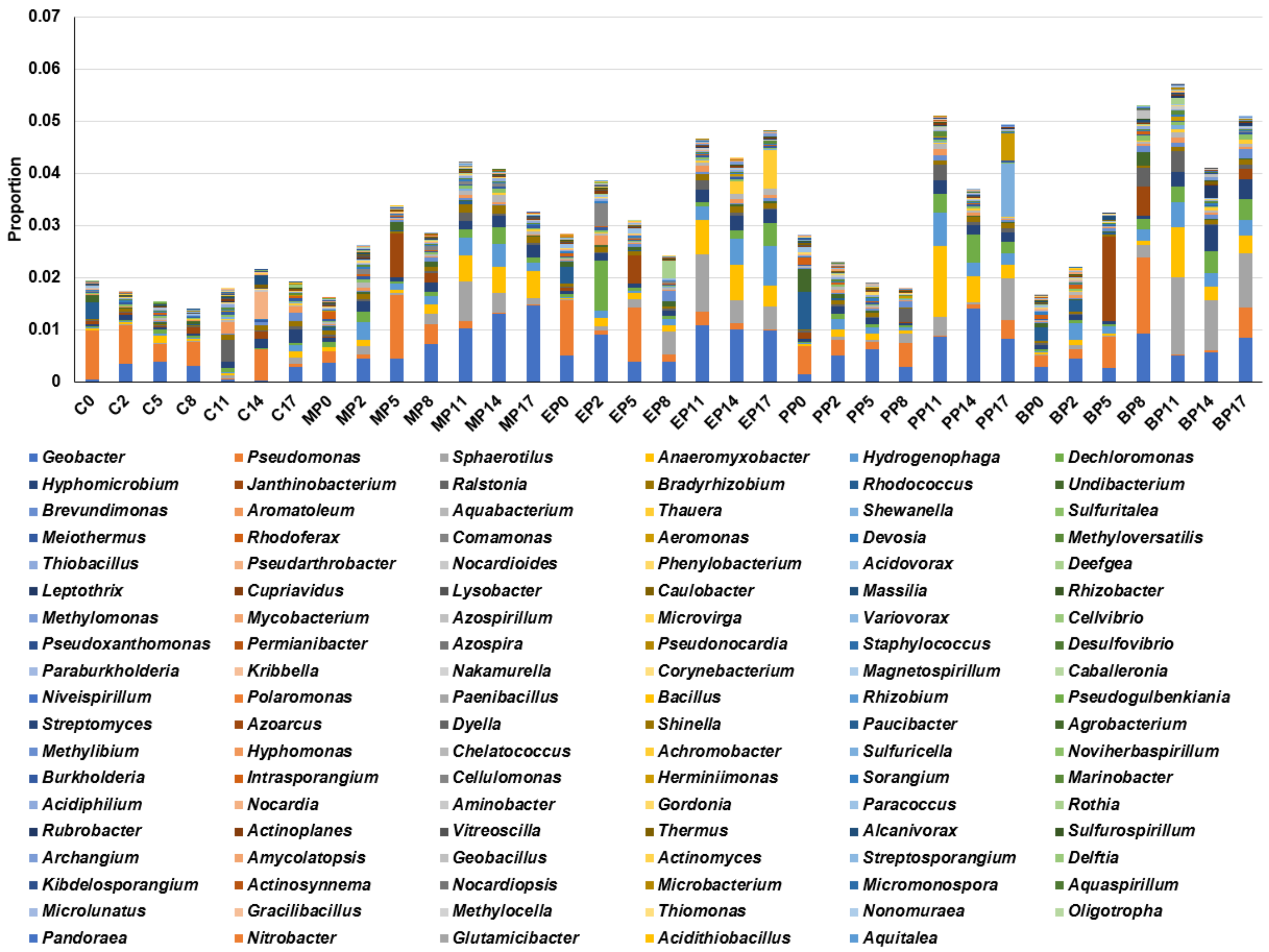

Figure S9. Dissimilatory nitrate reduction (M00530: nitrate => ammonia) associated microbial communities in the paraben treated river sediments. MP: methylparaben, EP: ethylparaben, PP: propylparaben, BP: butylparaben, C: control.

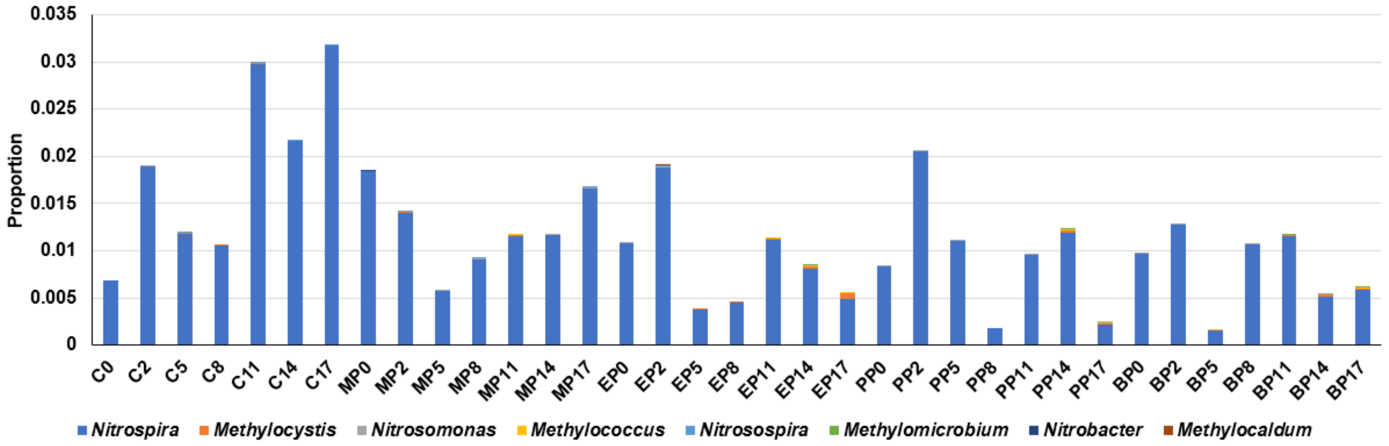

Figure S10. Nitrification (M00528: ammonia => nitrite) associated microbial communities in the paraben treated river sediments. MP: methylparaben, EP: ethylparaben, PP: propylparaben, BP: butylparaben, C: control.

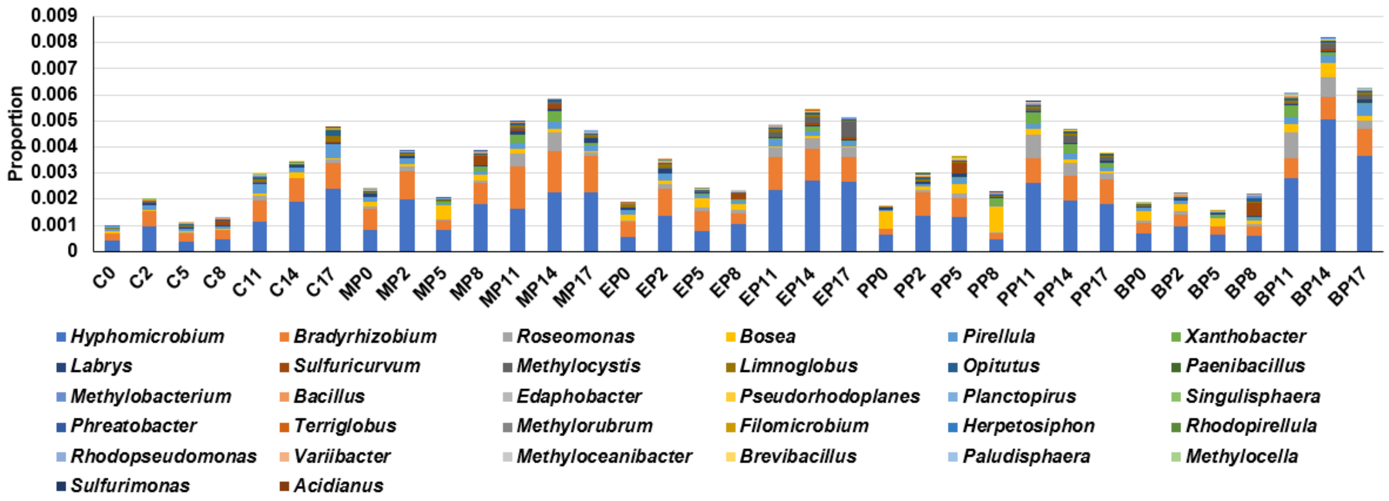

Figure S11. Assimilatory nitrate reduction (M00531: nitrate => ammonia) associated microbial communities in the paraben treated river sediments. MP: methylparaben, EP: ethylparaben, PP: propylparaben, BP: butylparaben, C: control.

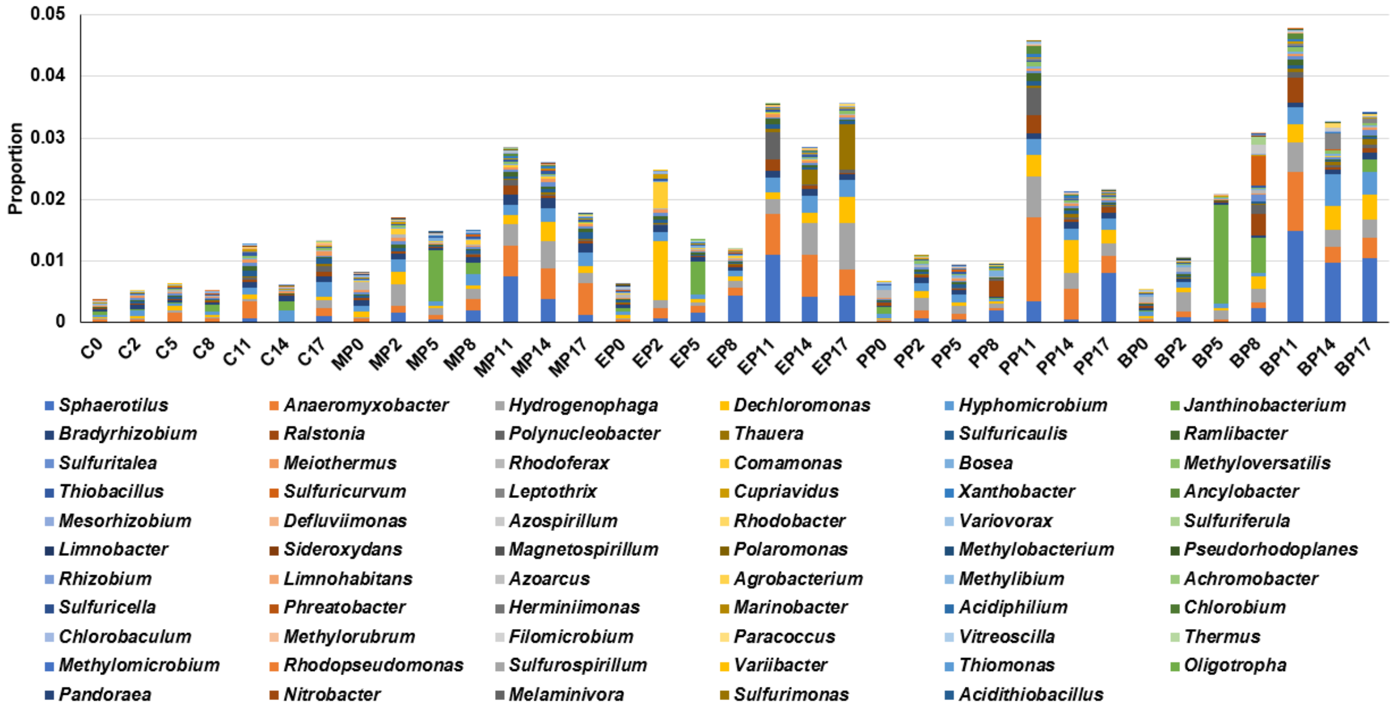

Figure S12. Thiosulfate oxidation (M00595: thiosulfate => sulfate) associated microbial communities in the paraben treated river sediments. MP: methylparaben, EP: ethylparaben, PP: propylparaben, BP: butylparaben, C: control.

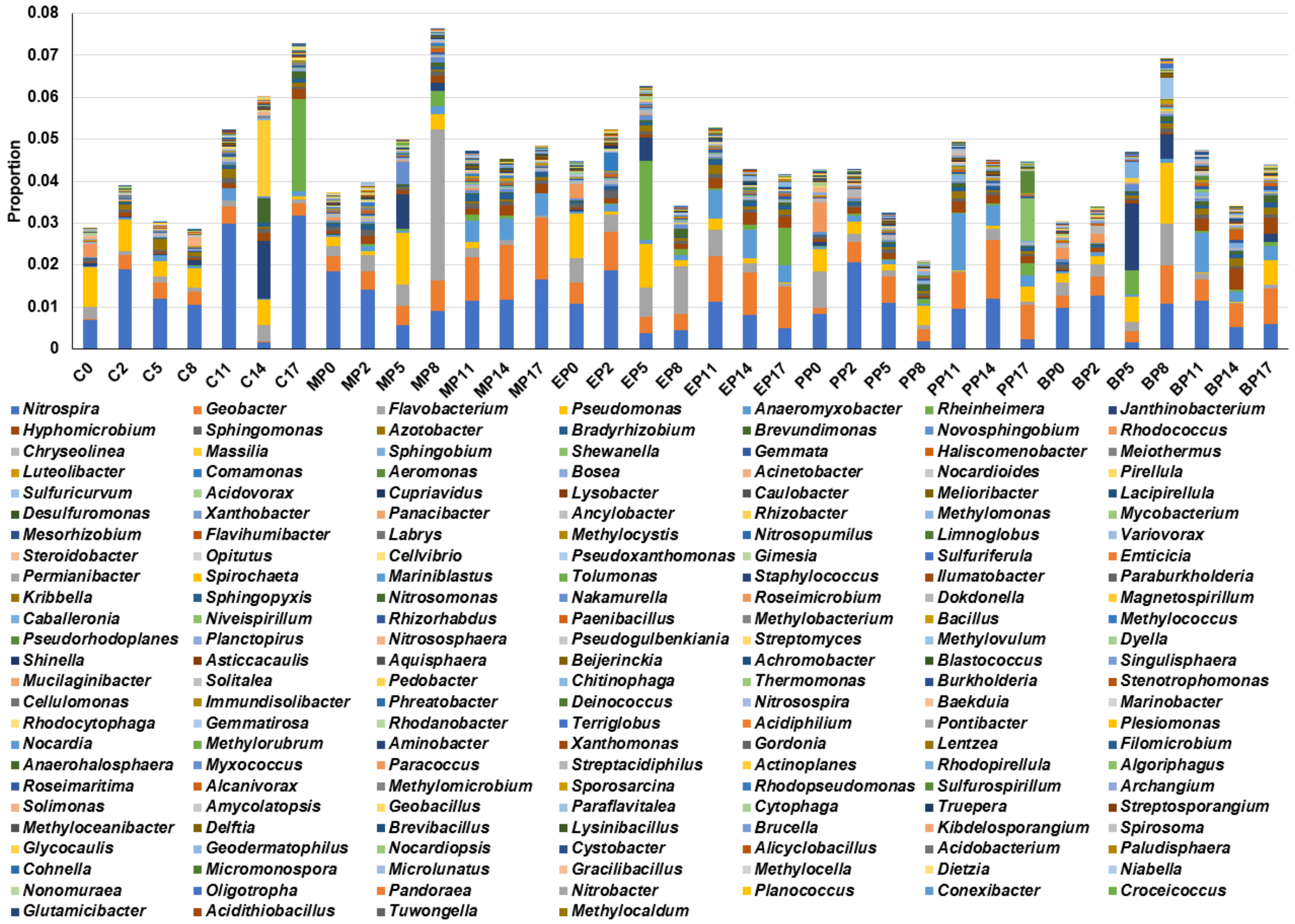

Figure S13. Assimilatory sulfate reduction (M00176: sulfate => H_2S) associated microbial communities in the paraben treated river sediments. MP: methylparaben, EP: ethylparaben, PP: propylparaben, BP: butylparaben, C: control.

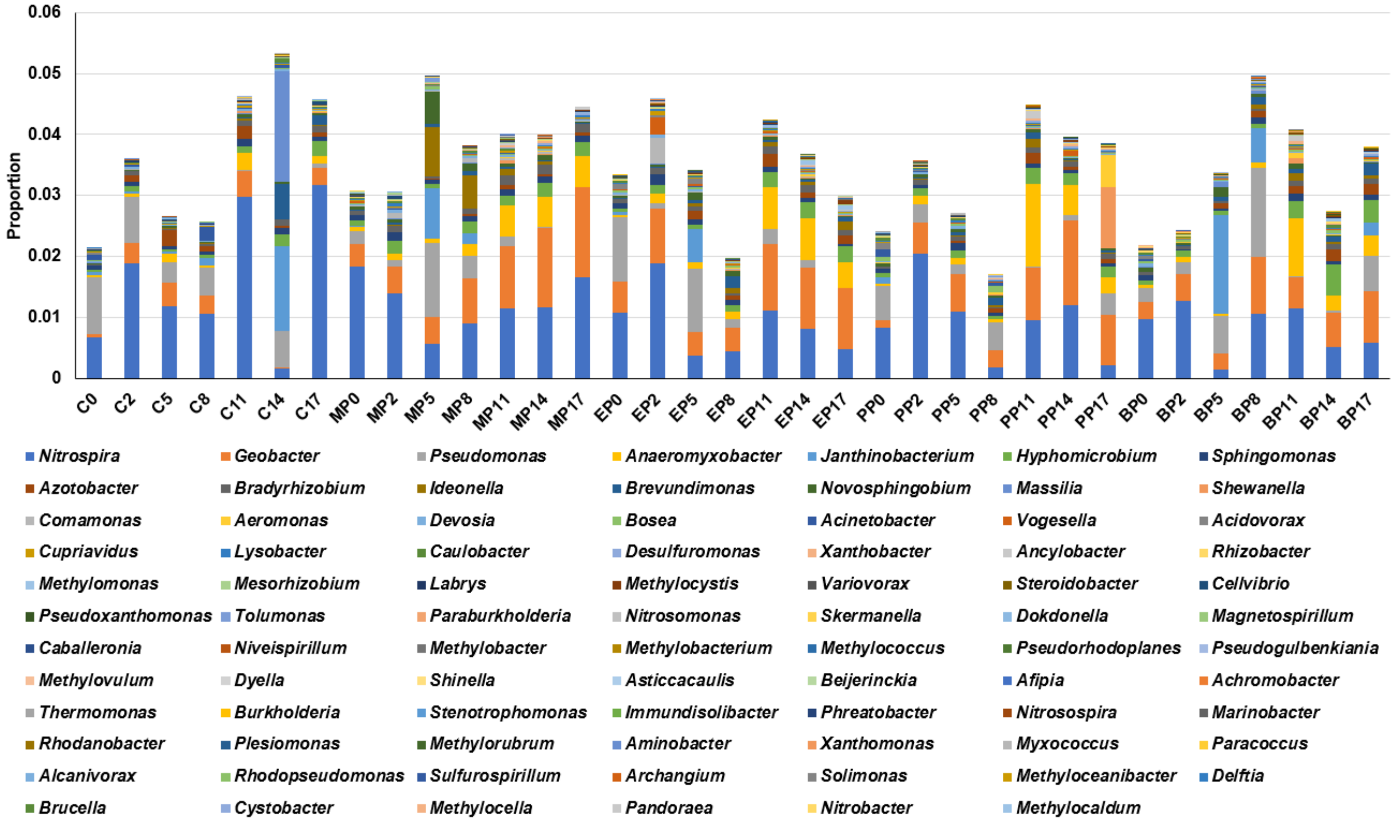

Figure S14. Sulfate-sulfur assimilation (M00616) associated microbial communities in the paraben treated river sediments. MP: methylparaben, EP: ethylparaben, PP: propylparaben, BP: butylparaben, C: control.

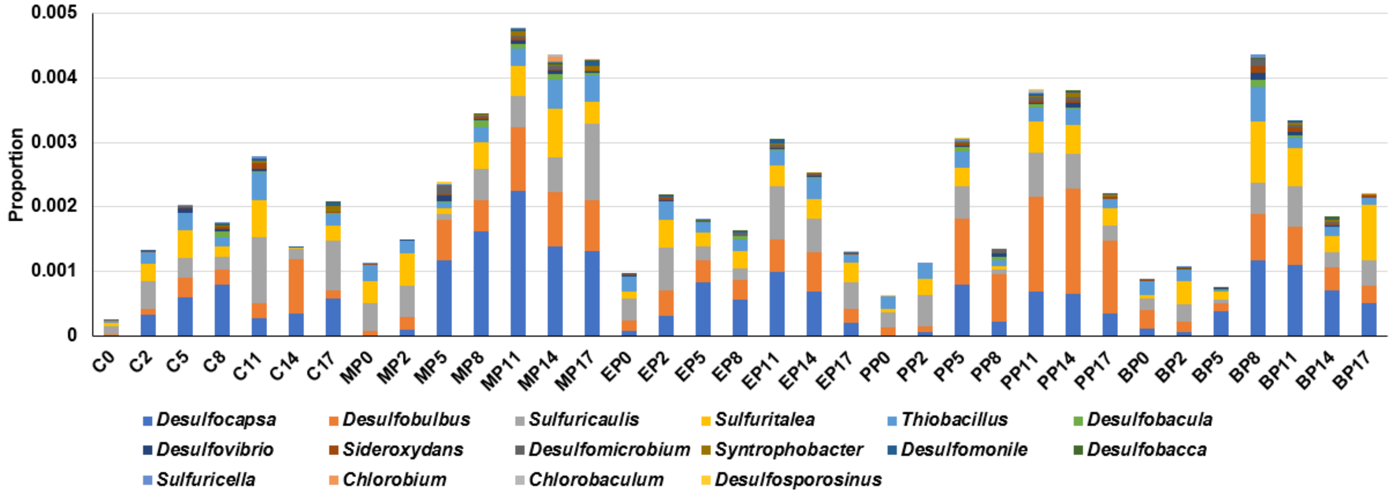

Figure S15. Dissimilatory sulfate reduction (M00596: sulfate => H_2S) associated microbial communities in the paraben treated river sediments. MP: methylparaben, EP: ethylparaben, PP: propylparaben, BP: butylparaben, C: control.

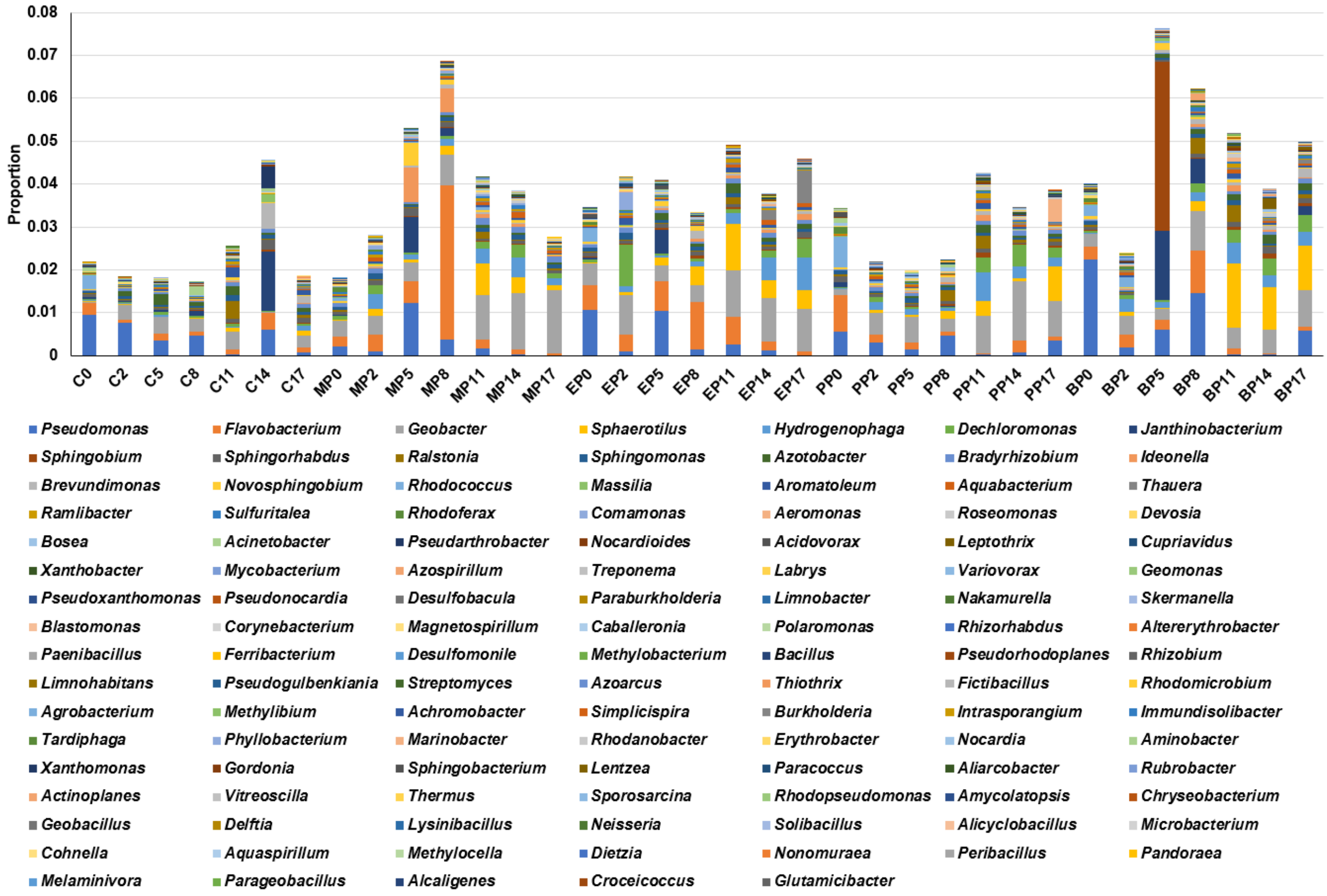

Figure S16. Microbial genera associated with xenobiotics degradation bacteria in the paraben treated river sediments. MP: methylparaben, EP: ethylparaben, PP: propylparaben, BP: butylparaben, C: control.

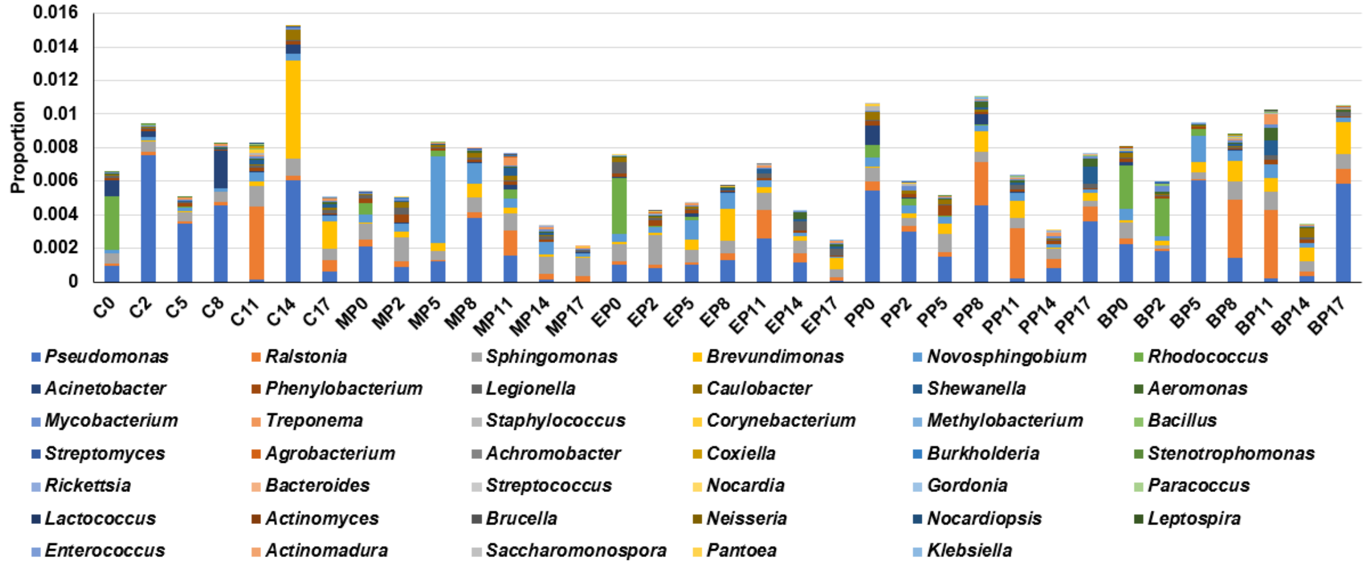

Figure S17. Proportions of microbial genera with potential pathogenic bacteria in the paraben treated river sediments. MP: methylparaben, EP: ethylparaben, PP: propylparaben, BP: butylparaben, C: control.

Table S1. Target compounds used in this study.

Target Compounds	abbreviation	CAS No	Formula
Methylparaben	MP	99-76-3	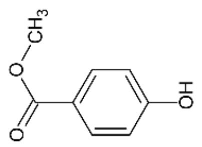
Ethylparaben	EP	120-47-8	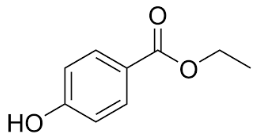
Propylparaben	PP	94-13-3	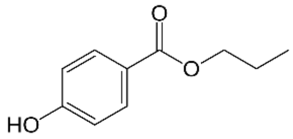
Butylparaben	BP	94-26-8	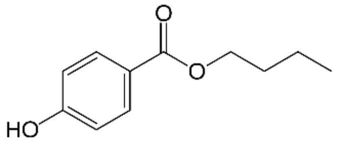

Table S2. Antibiotics used in this study.

Antibiotics	abbreviation	CAS No	Formula
Penicillin	Pen	113-98-4	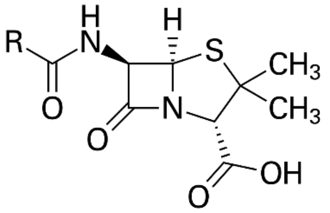
Tetracycline	Tet	60-54-8	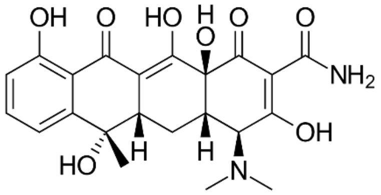
Sulfamethoxazole	Sul	723-46-6	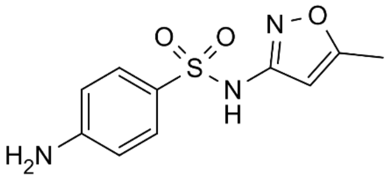
